# Watching liquid droplets of TDP-43_CTD_ age by Raman spectroscopy

**DOI:** 10.1016/j.jbc.2021.101528

**Published:** 2021-12-23

**Authors:** Sydney O. Shuster, Jennifer C. Lee

**Affiliations:** Laboratory of Protein Conformation and Dynamics, Biochemistry and Biophysics Center, National Heart, Lung, and Blood Institute, National Institutes of Health, Bethesda, Maryland, USA

**Keywords:** TDP-43, liquid–liquid phase separation, liquid droplets, amyloid, Raman spectroscopy, FRAP, TEM, CTD, C-terminal domain, FRAP, fluorescence recovery after photobleaching, LLPS, liquid–liquid phase separation, NHLBI, National Heart, Lung, and Blood Institute, TDP-43, TAR DNA-binding protein 43, TDP-43_CTD_, CTD of TDP-43, TEM, transmission electron microscopy, TEV, tobacco etch virus, ThT, thioflavin-T

## Abstract

Liquid–liquid phase separation (LLPS) is a biological phenomenon wherein a metastable and concentrated droplet phase of biomolecules spontaneously forms. A link may exist between LLPS of proteins and the disease-related process of amyloid fibril formation; however, this connection is not fully understood. Here, we investigated the relationship between LLPS and aggregation of the C-terminal domain of TAR DNA-binding protein 43, an amyotrophic lateral sclerosis–related protein known to both phase separate and form amyloids, by monitoring conformational changes during droplet aging using Raman spectroscopy. We found that the earliest aggregation events occurred within droplets as indicated by the development of β-sheet structure and increased thioflavin-T emission. Interestingly, filamentous aggregates appeared outside the solidified droplets at a later time, suggestive that amyloid formation is a heterogeneous process under LLPS solution conditions. Furthermore, the secondary structure content of aggregated structures inside droplets is distinct from that in *de novo* fibrils, implying that fibril polymorphism develops as a result of different environments (LLPS *versus* bulk solution), which may have pathological significance.

Interests in phase separation of amyloidogenic proteins have intensified recently as key features in liquid–liquid phase separation (LLPS)—low sequence complexity and conformational disorder—are also prevalent in amyloid formation (1, 2, 3). A number of pathological amyloids, including tau and α-synuclein, have been shown to phase separate and form liquid droplets *in vitro* (4, 5). Furthermore, TAR DNA-binding protein 43 (TDP-43) and fused in sarcoma protein, proteins associated with phase-separated compartments (*e.g.*, stress granules) in cells (6, 7), also have amyloid-forming domains (7, 8). The relationship between phase separation, protein aggregation, and disease remains to be elucidated. A prevailing hypothesis suggests that protein droplets could serve as loci of aggregation because of the hyperconcentrated pool of proteins (5, 6). This is evidenced by the observation that both phase-separated structures *in vitro* and *in vivo* can lose fluidity over time, preceding aggregation (5, 9, 10). Of note, recent *in vitro* work has suggested that TDP-43 amyloid aggregates can directly emerge from droplets as visualized by atomic force microscopy on dried samples, but how this transition occurs remains ill defined (11). Thus, it is essential to evaluate protein conformation state(s) inside droplets and to monitor how they change with time to determine a possible mechanistic connection to amyloid formation.

Here, we investigated conformational changes of the C-terminal domain (CTD) of TDP-43 (TDP-43_CTD_) during the aging process of droplets with spatial resolution by pairing a Raman spectrometer with an inverted microscope. TDP-43_CTD_ was chosen because its phase separation and aggregation into amyloid fibrils have been established (8, 11, 12). Specifically, a TDP-43_CTD_ mutant (W334F/W385F/W412F, referred to as W_free_) was used because of its improved solubility and purification yield (Fig. 1*A*). Raman spectroscopy was utilized because of experimental simplicity; it is an intrinsic (*i.e.*, probe free) measurement and provides direct information on protein secondary structure, in which α-helix, β-sheet, and disordered regions exhibit characteristic amide backbone frequencies (13, 14).

**Figure 1:**
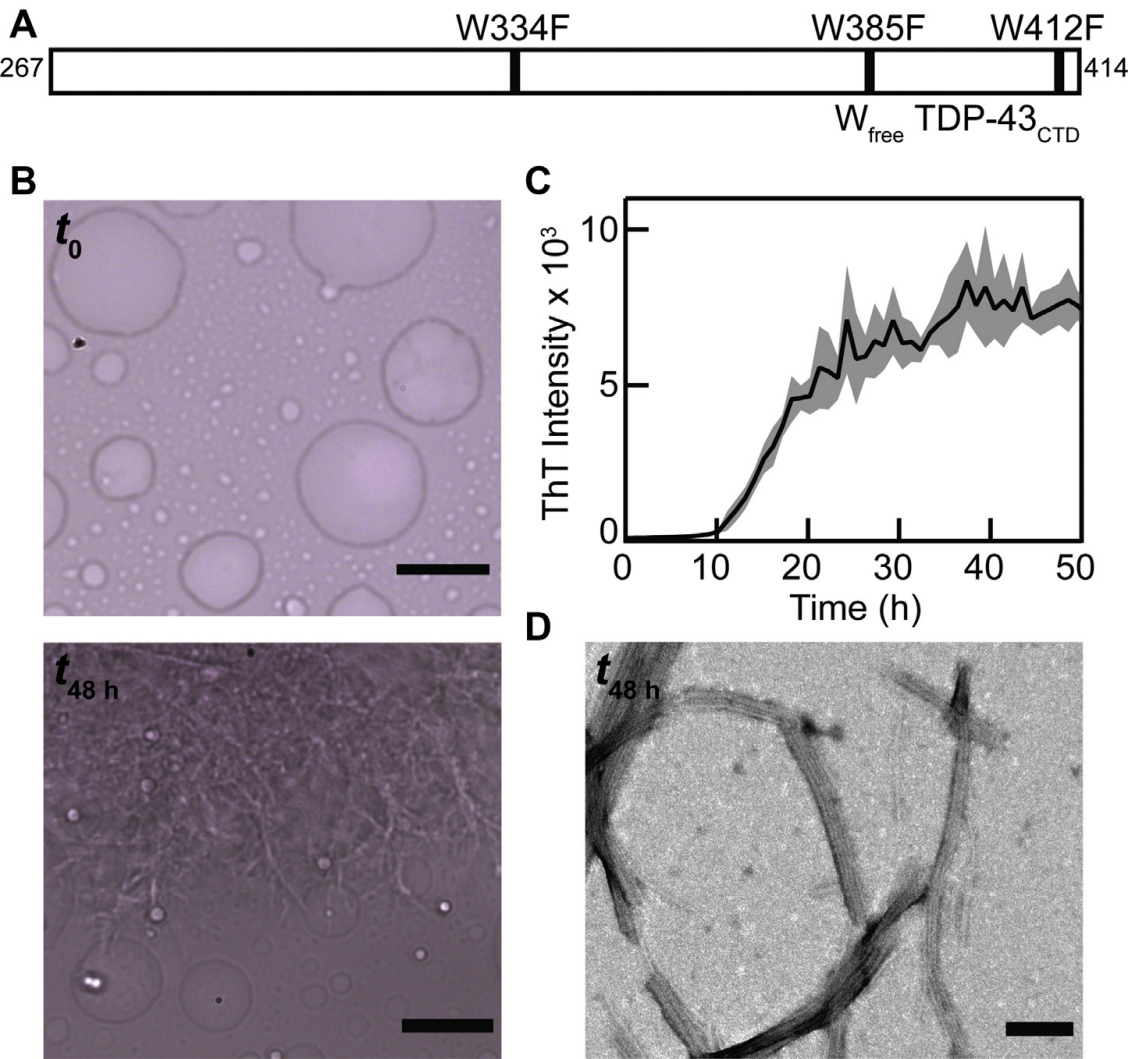
**TDP-43**_**CTD**_**phase separates and forms amyloid fibrils.***A*, schematic representation of the TDP-43_CTD_ construct (W_free_) used in this study, indicating three W-to-F mutations. *B*, bright-field images of W_free_ before (*t*_0_, *top*) and after 48 h of incubation (*t*_48 h_, *bottom*) at 22 °C (100 μM in 10 mM NaPi, 200 mM NaCl, pH 7.4 buffer). The scale bars represent 10 μm. *C*, aggregation kinetics of W_free_ monitored by ThT fluorescence ([W_free_] = 100 μM and [ThT] = 5 μM in 10 mM NaPi, 200 mM NaCl, and pH 7.4 buffer at 22 °C). *Line* and *shading* represent the mean and standard deviation, respectively (n = 3). *D*, representative TEM image of W_free_ after 48 h of incubation at 22 °C. The scale bar represents 100 nm. TDP-43_CTD_, C-terminal domain of TAR DNA-binding protein 43; TEM, transmission electron microscopy; ThT, thioflavin T.

## Results and discussion

LLPS of W_free_ was initiated by buffer exchange; droplets are evident immediately using bright-field microscopy (Fig. 1*B*, *top*). Upon incubation, the protein exhibits a canonical sigmoidal aggregation curve as evaluated by thioflavin-T (ThT), an amyloid-specific fluorophore (Fig. 1*C*) (15). Postaggregation at 48 h, bright-field (Fig. 1*B*, *bottom*) images show the persistence of droplets along with large fibrous aggregates, and transmission electron microscopy (TEM; Fig. 1*D*) reveals the existence of amyloid fibrils. Clearly, W_free_ phase separates and aggregates into amyloid fibrils under the same solution conditions. However, it is unclear what role LLPS plays in this amyloid formation process.

**Figure 2:**
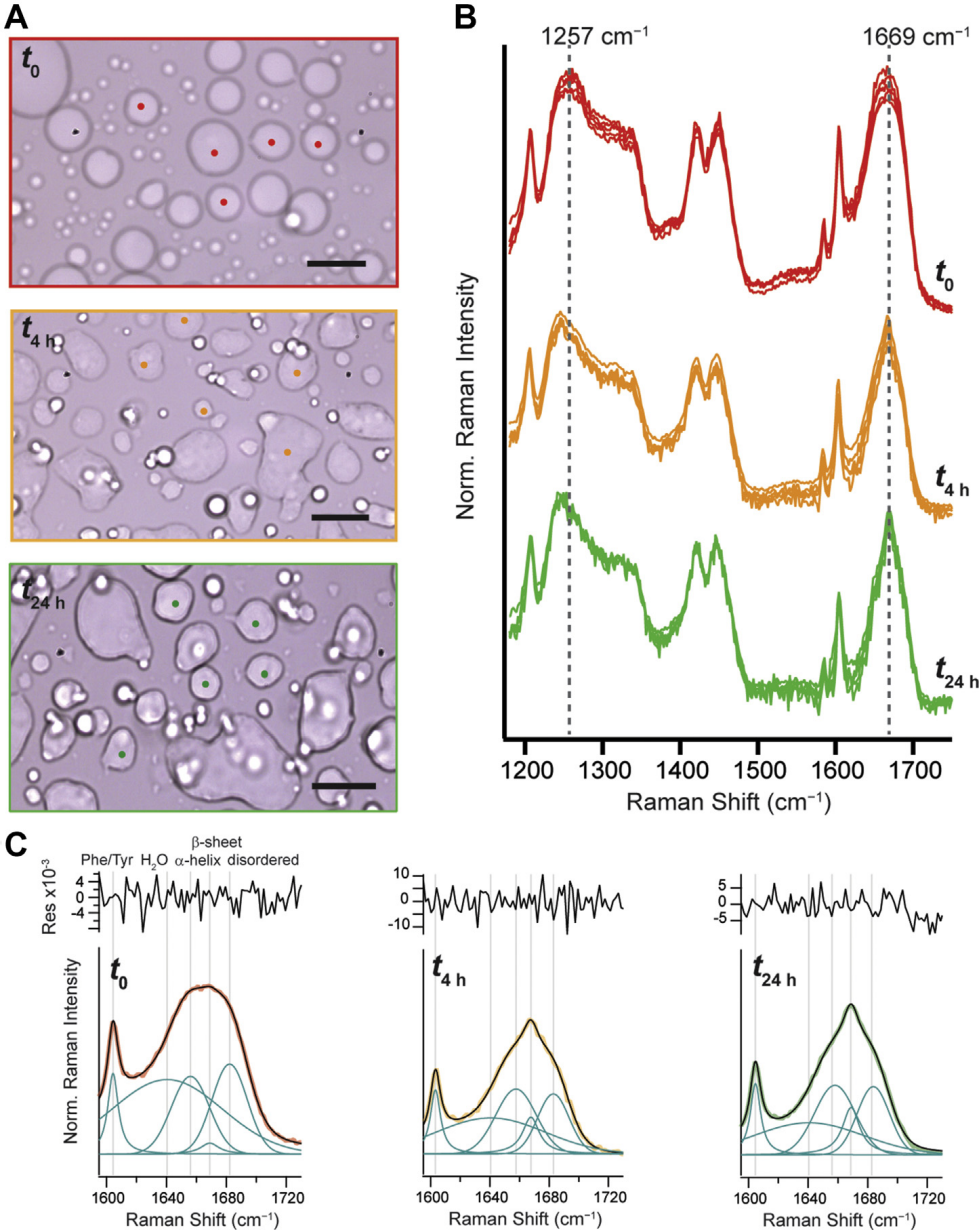
**TDP-43**_**CTD**_**droplet aging monitored by Raman spectroscopy.***A*, bright-field images and (*B*) Raman spectra of W_free_ before (*t*_0_, *red curves*), after 4 h (*t*_4 h_, *orange curves*), and 24 h (*t*_24 h_, *green curves*) of incubation at 22 °C (n = 5). Locations where the Raman spectra were collected are denoted by the same color spots in *A*. The scale bars represent 10 μm. [W_free_] =100 μM in 10 mM NaPi, 200 mM NaCl, and pH 7.4. Spectra were collected with 15 accumulations of integration time of 3 s. *Dashed lines* are drawn to provide guides of spectral evolution as a function of time. Spectra are offset for clarity and normalized to the Phe breathing peak (1003 cm^−1^) for comparison. *C*, fits of the amide-I band region. Data were averaged and colored as in *B*. Fits and individual peak components are shown in *black* and *cyan*, respectively, where the *gray lines* denoted peak center positions, and assignments are as indicated. Residuals are also shown above. Fitting parameters and results are reported in Tables S1 and S2. Additional dataset is shown in Fig. S2. TDP-43_CTD_, C-terminal domain of TAR DNA-binding protein 43.

To address this question, protein secondary structural changes of droplets were measured by Raman spectroscopy. Phase-separated droplets are first visualized by bright-field microscopy. Then, Raman spectra are measured at selected locations and monitored up to 48 h. Bright-field images taken immediately (*t*_0_) and after 4 (*t*_4 h_) and 24 h (*t*_24 h_) indicate that droplet distortions appear at 4 h and become pervasive at 24 h (Fig. 2*A*), highlighting a time-dependent transformation. Correspondingly, Raman spectra collected from droplets show unique features (Fig. 2*B*). At *t*_0_, the amide-I band is broad, which is characteristic of complex mixture of secondary structural components (16). By 4 h, however, a sharper component emerges at 1669 cm^−1^, consistent with β-sheet structure development (13, 16), which increases modestly in the following 20 h. This increase in β-sheet character as the droplets age is recapitulated by a red-shifted amide-III peak.

To quantify the differences, the amide-I regions were decomposed into individual peaks (Table S1 and Fig. 2*C*): aromatic side chains (∼1604 cm^−1^), water (Fig. S1), and secondary structure components—α-helix (∼1657 cm^−1^), β-sheet (∼1669 cm^−1^), and disordered (∼1683 cm^−1^). At *t*_0_, the protein secondary structure inside the droplets is composed of 45% α-helical, 5% β-sheet, and 50% disordered conformation (Table S2). Previous Raman studies on phase-separated proteins also have indicated the presence of disordered conformation for the low complexity domain of fused in sarcoma and ataxin-3 (17, 18). Helical structure for TDP-43_CTD_ droplets has been previously reported (12, 19); however, the Raman data indicate a greater helical content than would be expected for the short transient helix (<10%) characterized by NMR.

Upon aging for 4 h, conformations with increased β-sheet structure are observed with some decrease in the disordered component but no significant change in helical content (Table S2). Interestingly, a loss of water content is also evident. Formation of β-sheet structure suggests that protein aggregation is occurring, which is supported by the observation of dehydration. In the following 20 h, there were insignificant spectroscopic changes. These trends were consistent across independent experiments (Fig. S2 and Table S3). The presence of α-helical conformation is unexpected as TDP-43_CTD_ aggregates are characterized to be β-sheet rich (8, 20). The stabilization of the polypeptide structure inside the droplets by 4 h is also intriguing as this corresponds to the lag phase of aggregation.

**Figure 3:**
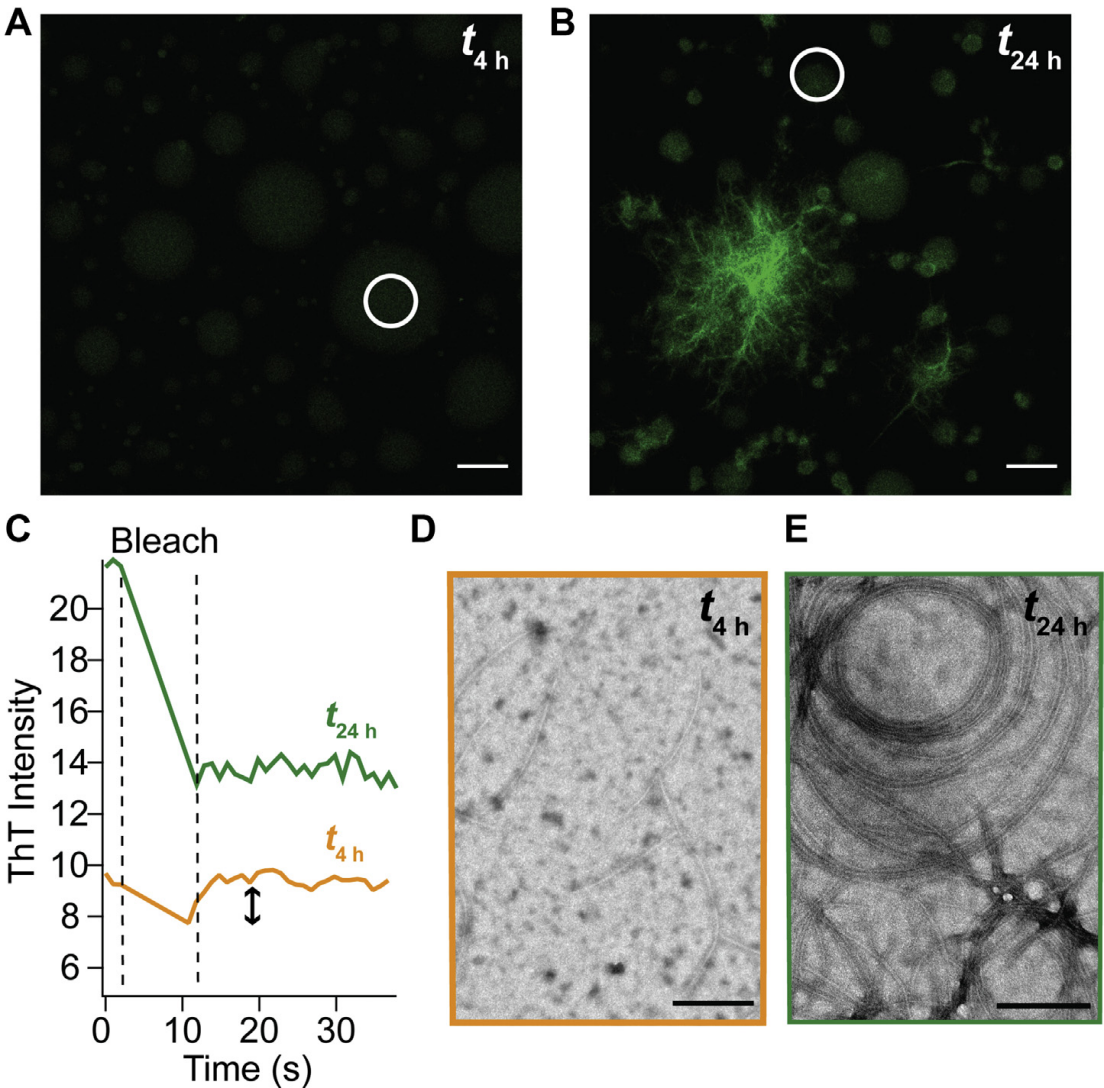
**FRAP reveals differences between TDP-43**_**CTD**_**samples aged at 4 and 24 h.** Confocal fluorescence images of W_free_ after (*A*) 4 h and (*B*) 24 h of incubation at 22 °C ([W_free_] =100 μM in 10 mM NaPi, 200 mM NaCl, pH 7.4) in the presence of ThT (5 μM). Same intensity scales are shown for both images. The scale bars represent 10 μm. *C*, ThT fluorescence recovery was monitored postbleaching of ThT-stained droplets at 4 and 24 h. Specific regions are indicated by *white circles* in *A* and *B*. Additional data are shown in Fig. S3*A*. TEM images of W_free_ after (*D*) 4 h and (*E*) 24 h of incubation at 22 °C. The scale bars represent 200 nm. FRAP, fluorescence recovery after photobleaching; TDP-43_CTD_, C-terminal domain of TAR DNA-binding protein 43; TEM, transmission electron microscopy; ThT, thioflavin T.

To interrogate this further, we turned to confocal fluorescence microscopy and fluorescence recovery after photobleaching (FRAP) experiments using ThT to provide information on whether amyloid aggregation and solidification has occurred, respectively. There is obvious structural maturation in which a higher ThT intensity is measured at 24 h, consistent with amyloid formation (Fig. 3, *A* and *B*). Upon photobleaching, the 4 h droplets quickly recover (Figs. 3*C* and S3), indicating freely diffusing fluorophores in exchange with the bulk solution. In contrast, there is sequestered ThT at 24 h, suggestive of solidification. Notably, emissive filamentous aggregates are also now apparent at 24 h. These results show that the droplets are evolving from 4 to 24 h, even though the protein secondary structures within them remain similar.

TEM characterization at the ultrastructural level also indicates differences between the two times. At 4 h, only a few filamentous aggregates are observed (Fig. 3*D*), which become numerous, larger, bundled fibrils by 24 h (Fig. 3*E*). We note that these filaments at 4 h are not associated with droplets and appear similar to the intermediate fibrils previously observed in non–phase-separating conditions (*i.e.*, no salt) for TDP-43_CTD_ (8). This led us to question whether the aggregation process that leads to large fibril bundles visible in the bright-field and confocal fluorescence images is in fact distinct from droplet solidification.

**Figure 4:**
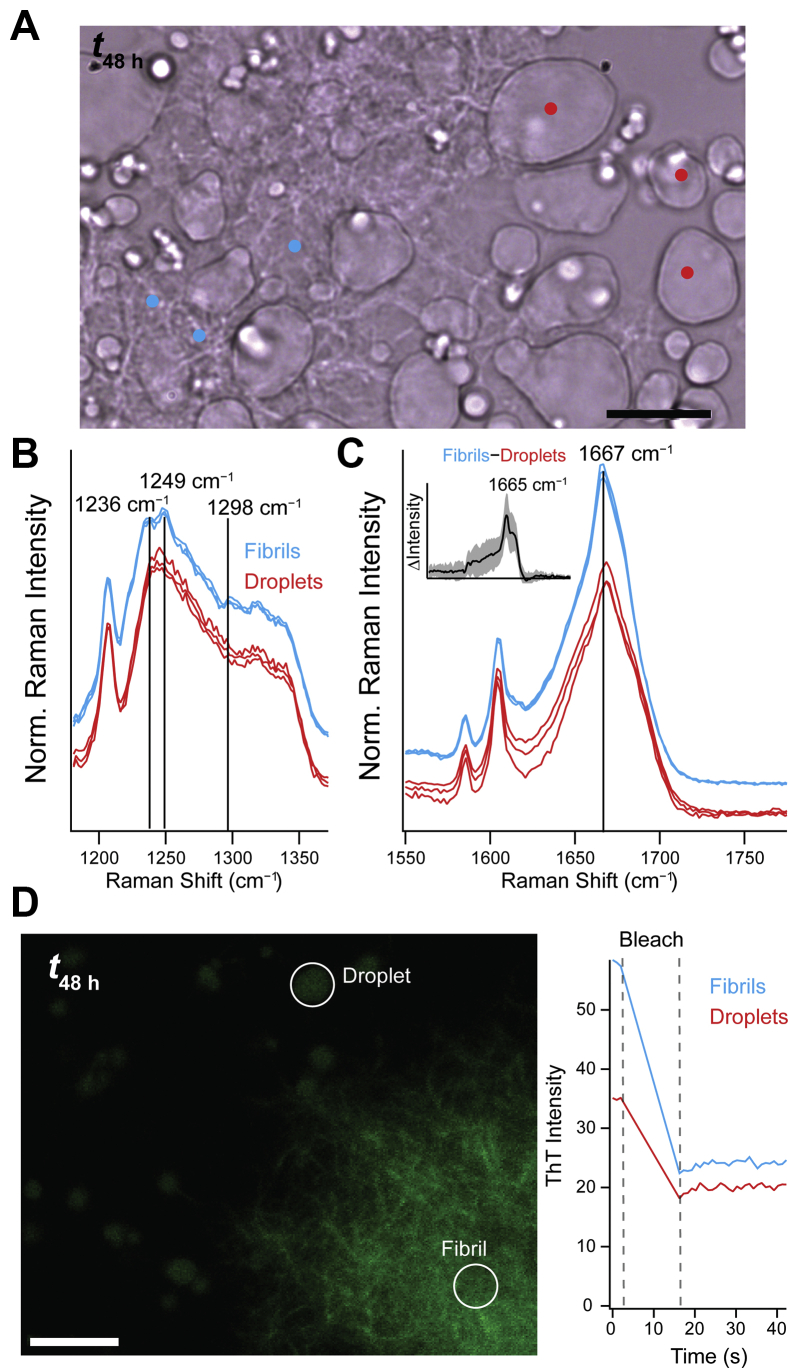
**Raman spectra reveal structural differences between aged droplets and fibrils at 48 h.***A*, bright-field image of W_free_ after 48 h of incubation at 22 °C ([W_free_] =100 μM in 10 mM NaPi, 200 mM NaCl, pH 7.4). *Colored dots* indicate locations where Raman spectra were collected. *Cyan* and *red* indicate fibril *versus* droplet morphology, respectively. The scale bar represents 10 μm. Raman spectra in the (*B*) amide-III and (*C*) amide-I regions. Spectra are offset for clarity and normalized to the Phe breathing peak (1003 cm^−1^) for comparison. *Inset*, difference spectrum (fibril–droplet) of the amide-I region. *Line* and *shading* represent the mean and standard deviation, respectively (n = 3). *Guidelines* are drawn for reference. Full spectra and fit results can be found in Fig. S4 and Table S4. Additional data are shown in Fig. S5. *D*, confocal fluorescence image of W_free_ after 48 h of incubation at 22 °C in the presence of ThT (5 μM, *left*). The scale bar represents 10 μm. Representative droplets (*red*) and fibrils (*cyan*) were bleached in a circular region of interest (indicated by *circles*), and fluorescence recovery was monitored postbleaching (*right*). Additional data are shown in Fig. S3*B*. ThT, thioflavin T.

To test this hypothesis, we once again turned to Raman spectroscopy to delineate any spectral differences between the fibrous aggregates and the droplets at 48 h. Because both solidified droplets and filamentous aggregates (referred to as fibrils) are present in large numbers, measurements can be made within droplets and fibrils in the same field of view (Fig. 4*A*). Both the amide-III (Fig. 4*B*) and amide-I (Fig. 4*C*) regions display distinctive spectral features for droplets and fibrils. In the amide-III region, there is a single peak in the droplets, which resolves into two peaks at 1236 and 1249 cm^−1^ in the fibrils. A new peak also appears at 1298 cm^−1^ in the fibrils, supportive of different conformations. The difference spectrum of the amide-I region (Fig. 4*C*, *inset*) highlights an enhancement of the β-sheet peak (1665 cm^−1^) in the fibrils as compared with the droplets. Based on the fits, there is more than a twofold increase (24–60%) of the β-sheet component along with a comparable decrease (39–13%) of the α-helical component in the fibrils (Fig. S4 and Table S4). This change in secondary structural composition along with a small red-shift of the amide-I band (∼2 cm^−1^) is highly reproducible (Fig. S5 and Table S5), substantiating that the fibrils formed in bulk solution are distinct from the aggregates inside droplets. While it is plausible that these differences could represent incomplete aggregation in droplets compared with fibrils, the respective FRAP data would indicate otherwise, as neither the droplets or fibrils have observable exchange with the bulk solution (Fig. 4*D*). We cannot, however, rule out interplay between the two species (*e.g.*, nucleation off the side of a hardened droplet) as they occur in the same area with fibrils appearing near, around, and even on top of droplets. Moreover, droplets may also have divergent paths, possibly with some droplets remaining liquid and transforming into fibrils. However, all droplets examined here solidified. Interestingly, seeded aggregation of TDP-43_CTD_ was shown to be delayed under LLPS conditions (21), suggestive of underlying structural incompatibility of the aggregates. This then could explain the observed differences in ThT activities of the aggregates in droplets and fibrils, reflecting fibril polymorphism as reported in other amyloids (22).

In summary, this work demonstrates that Raman spectroscopy is a simple and powerful approach to study protein conformational changes in LLPS. We have directly observed droplet maturation of TDP-43_CTD_, offering detailed structural information with spatial context, which would be otherwise obscured by bulk measurements. TDP-43_CTD_ in droplets initially contain a mixture of α-helical and disordered segments, but as time progresses, β-sheet structure develops, consistent with amyloid formation within droplets. In addition, a slower appearance of filamentous aggregates is seen outside the droplets, after which time the solidified droplets are not in exchange with the solution. These results suggest that TDP-43_CTD_ aggregation in phase-separating conditions is heterogeneous, with aggregation occurring first within droplets, followed by the formation of amyloids in solution from the remaining pool of monomers. Although both types of aggregates are amyloid in nature, aged TDP-43_CTD_ structures in droplets and fibrils are clearly distinct, and likely separate polymorphs based on their unique Raman spectroscopic features and ThT intensity differences. As polymorphism is implicated in some disease phenotypes such as α-synuclein in Parkinson's, dementia with Lewy bodies, and multiple system atrophy (23, 24), this raises intriguing questions about whether different TDP-43-aggregated structures (if any) could be involved in the progression of amyotrophic lateral sclerosis and frontotemporal dementia (25). There is future potential for impact of similar Raman studies, not only with other proteins but also by coupling tools from chemical biology such as native chemical ligation and unnatural amino acid incorporation to gain residue-specific information on LLPS. Finally, since Raman spectroscopy is compatible with cellular imaging (26, 27) this technique could be developed to evaluate the chemical composition of biomolecules in phase-separated compartments and their dynamic assembly in cells.

## Experimental procedures

### Reagents

Unless otherwise noted, all reagents used were purchased from Sigma–Aldrich.

### Recombinant protein expression and purification

TDP-43_CTD_ plasmid was a gift from Nicolas Fawzi (Addgene; plasmid #98669) (12). The construct consisted of an N-terminal Thio6 expression tag, histidine tag, tobacco etch virus (TEV) cleavage site, followed by residues 267 to 414. Trp-to-Phe mutations (residues 334, 385, and 412) were done *via* a QuikChange site-directed mutagenesis kit (Agilent) using the following primers: 5′-GCAAGAGCTCCGGTTTTGGCATGT AACTCG-3′ (W412F), 5′-GGTGCGGCAATCGGCTTTGGTAGCGCAAGCAATG-3′ (W385F), and 5′-GCGCTGCAGT CTAGCTTTGGTATGATGGGCATG-3′ (W334F). WT or W334F/W385F/W412F (W_free_) TDP-43_CTD_ plasmid was transformed into *Escherichia coli* BL21 (DE3) (Invitrogen), expressed and purified as previously described (8). TEV-cleaved proteins contain an N-terminal overhang consisting of residues GHM and were assessed with >95% purity by SDS-PAGE and LC–MS. Measured mass was 14773.1 Da for W_free_. Protein was concentrated to ∼50 to 250 μM using 3 kDa centrifugal filters (Amicon), aliquoted, and rapidly frozen in liquid nitrogen. Protein was stored at −80 °C until use. All buffers were filtered (0.22 μm).

### Aggregation kinetics

Protein solutions were thawed on ice and buffered exchanged into 10 mM NaPi, 200 mM NaCl, pH 7.4 using PD-10 columns (Cytiva). Protein concentrations were determined on a Cary 300 Series UV–Vis Spectrometer (Agilent Technologies) using molar absorptivity at 280 nm (ε_280 nm_ [W_free_] = 1490 M^−1^ cm^−1^) reported by ProtParam (ExPasy). Because of the lower ε_280 nm_, W_free_ concentration was also confirmed using a standard bicinchoninic acid assay (Pierce). Reactions of 70 μl were performed in clear 384-well polypropylene flat bottom plates (Greiner Bio-One; catalog no.: 781261), sealed with a MicroAmp optical adhesive film (Thermo Fisher Scientific), and monitored using a SPARK Multimode Microplate reader (Tecan) maintained at 22 °C. ThT fluorescence (excitation and detection wavelengths were 415 and 480 nm, respectively) was measured in intervals of 1 h The microplate was shaken linearly at 6 mm for 5 s in between each read. Three independent experiments with five technical replicates were performed. Reproducibility and consistency were checked using two different protein expressions and preparations.

### Raman spectroscopy

Raman spectra were collected using a home-built instrument as previously described (28). The bright-field image was collected using a USB 2.0 camera (iDS; UI-1220-C). Chambers were constructed from a microscope slide (VWR; catalog no.: 16004-422), imaging chamber (Grace Bio-Labs CoverWell; PCI-A-0.5; 20 mm diameter × 0.8 mm depth), and a #1 coverslip (22 mm square; Corning; catalog no.: 2865-22). Chambers were filled with 200 μl of protein sample taken immediately from desalting (described previously). Protein concentrations ranged from 80 to 120 μM. Immediately upon desalting (∼4 min), the solution is turbid, indicating that phase separation has occurred. In between time points, chambers were kept with the coverslip side down and maintained at 22 °C. Representative spectra are shown with each collection constituting 15 to 25 accumulations with an integration time of 3 s. Data were processed by buffer subtraction using LabSpec 6 software (Horiba Scientific). For comparison, spectra were baseline subtracted at 2000 cm^−1^ and normalized to the phenylalanine breathing mode (1003 cm^−1^), which is insensitive to protein conformation, using Igor 7.06 (Wavemetrics). Amide-I peaks were fit using the multi-fit 2.2 package in Igor 7.06 (Wavemetrics) using Lorentzian and Gaussian functions and a constant baseline. Five peaks were used (Table S1). They are as follows: peak 1, a Lorentzian function with an initial guess of 1603 cm^−1^, representing aromatic peaks of Phe and Tyr; peak 2, a Gaussian function held at 1640.5 cm^−1^ and an full width at half maximum of 86.59 cm^−1^, representing the bending mode of water; peak 3, a Gaussian function with an initial guess of 1655 cm^−1^, representing an α-helical structure component; peak 4, a Lorentzian function with an initial guess of 1665 cm^−1^, representing a β-sheet structure component; and peak 5, a Gaussian function with an initial guess of 1685 cm^−1^, representing a disordered structure component. The parameters for the Gaussian function representing water (peak 2) was determined by fitting a buffer spectra with a single peak and a cubic baseline subtraction to account for contribution from glass. In the case of the *t*_48 h_ fibril data, bounds were placed on the α-helical and disordered component locations in order to obtain the best fits. Lorentzian *versus* Gaussian was decided on which gave the best and most reproducible fits according to χ^2^ values.

### TEM

Samples were prepared by desalting as described previously and incubated in Eppendorf tubes in a benchtop incubator maintained at 22 °C and withdrawn at specified times. TEM images were collected on a JEOL JEM 1200 EXII microscope equipped with an XR-60 digital camera (Advanced Microscopy Techniques) operating at 80 kV (NHLBI Electron Microscopy Core). Grids were prepared by depositing 5 μl of solution onto a 400-mesh copper grid with a formvar/carbon film (Electron Microscopy Sciences) for 1 min. Excess solution was wicked away using grade 1 Whatman filter paper (GE Healthcare). Grids were washed once with filtered deionized water. Grids were then stained for 10 s using 5 μl of 1% (w/v) uranyl acetate, followed by wicking with filter paper. Finally, grids were dried at room temperature before collecting images.

### Confocal fluorescence microscopy and FRAP

Fluorescence images and FRAP experiments were acquired on a Zeiss 780 confocal microscope (NHLBI Light Microscopy Core) using a 63× oil immersion objective (numerical aperture, 1.4) using ZEN software (Zeiss). Chambers were constructed as described previously with the addition of 5 μM ThT. ThT fluorescence was excited by a 405 nm laser at 2% power and collected at 415 to 476 nm. Photobleaching was performed using 100% laser power for 6 s. Fluorescence intensity recovery was monitored for approximately 30 s. At each time point, at least five distinct spatial locations were measured. Images were analyzed with Fiji (29). All images were set to the same brightness/contrast settings in order to make intensity comparisons unless otherwise noted. No background subtraction was performed.

## Data availability

All data are available in the main text or the supporting information.

## Supporting information

This article contains supporting information.

## Conflict of interest

The authors declare that they have no conflicts of interest with the contents of this article.
