# Supplementary material for: Watching liquid droplets of TDP-43CTD age by Raman spectroscopy
Source: J Biol Chem. 2021 Dec 23;298(2):101528. doi: 10.1016/j.jbc.2021.101528 (PMC8784639; doi:10.1016/j.jbc.2021.101528)
Supplement: Supplemental Tables S1–S5 and Figures S1–S5 [file mmc1.pdf]

SI Appendix for

Watching liquid droplets of TDP-43$_{CTD}$ age by Raman spectroscopy

SO Shuster and JC Lee

Table S1. General fit parameters for amide-I region

Table S2. Fit results for Raman spectra shown in Fig. 2C

Table S3. Fit results for Raman spectra shown in Fig. S3

Table S4. Fits of Raman spectra shown in Fig. S4

Table S5. Fits of Raman spectra shown in Fig. S5

Figure S1. Fit of the water bend mode in the amide-I region

Figure S2. Additional data set and their fits of t_0, $t_{4\ h}$, and $t_{24\ h}$

Figure S3. Normalized FRAP data for $t_{4\ h}$, $t_{24\ h}$, and $t_{48\ h}$

Figure S4. Full Raman spectra and amide-I fits for droplets and fibrils after 48 h of incubation

Figure S5. Additional data set and their fits of droplets and fibrils at $t_{48\ h}$

Table S1. General fit parameters used for fitting amide-I band

Peak	Peak type	Location (cm^{-1})	Width (cm^{-1})	FWHM (cm^{-1})	Assignment
**1**	Lorentzian	unbounded	N/A	unbounded	Tyr/Phe
**2**	Gaussian	1640.5	52	N/A	Water bend
**3**	Gaussian	1650–1658	10–35	N/A	α-helix
**4**	Lorentzian	1665–1670	N/A	5–25	β-sheet
**5**	Gaussian	1680–1686	10–30	N/A	disordered

Table S2. Fits of Raman spectra shown in Fig. 2C

t_0						
**Peak**	Peak type	Location (cm^{-1})	FWHM (cm^{-1})	Raw Area	Percent of Total Area	Percent of secondary structure content
**1**	Lorentzian	1604.3	8.18	2.36	0.08	
**2**	Gaussian	1640.5	86.59	16.62	0.53	
**3**	Gaussian	1656.2	28.07	5.54	0.18	0.45
**4**	Lorentzian	1668.7	10.87	0.47	0.02	0.05
**5**	Gaussian	1681.2	26.35	6.11	0.20	0.50
**$t_{4\ h}$**						
**Peak**	Peak type	Location (cm^{-1})	FWHM (cm^{-1})	Raw Area	Percent of Total Area	Percent of secondary structure content
**1**	Lorentzian	1603.2	8.34	2.05	0.10	
**2**	Gaussian	1640.5	86.59	8.12	0.38	
**3**	Gaussian	1657.6	31.95	5.4	0.25	0.48
**4**	Lorentzian	1667.7	13.69	1.93	0.09	0.17
**5**	Gaussian	1682.8	24.97	3.9	0.18	0.35
$t_{24\ h}$						
**Peak**	Peak type	Location (cm^{-1})	FWHM (cm^{-1})	Raw Area	Percent of Total Area	Percent of secondary structure content
**1**	Lorentzian	1604.6	8.45	2.30	0.10	
**2**	Gaussian	1640.5	86.59	7.23	0.32	
**3**	Gaussian	1658	32.02	5.79	0.26	0.46
**4**	Lorentzian	1668.8	13.95	2.51	0.11	0.19
**5**	Gaussian	1683.7	25.72	4.56	0.20	0.35

Table S3. Fits of Raman spectra shown in Fig. S2

t_0						
**Peak**	Peak type	Location (cm^{-1})	FWHM (cm^{-1})	Raw Area	Percent of Total Area	Percent of secondary structure content
**1**	Lorentzian	1605.2	8.01	2.17	0.08	
**2**	Gaussian	1640.5	86.59	11.69	0.46	
**3**	Gaussian	1657.4	30.6	5.44	0.21	0.46
**4**	Lorentzian	1669.5	14.17	0.86	0.03	0.07
**5**	Gaussian	1683.3	27.6	5.58	0.22	0.47
**$t_{4\ h}$**						
**Peak**	Peak type	Location (cm^{-1})	FWHM (cm^{-1})	Raw Area	Percent of Total Area	Percent of secondary structure content
**1**	Lorentzian	1604.6	8.03	2	0.09	
**2**	Gaussian	1640.5	86.59	8.62	0.39	
**3**	Gaussian	1657.6	30.35	4.95	0.22	0.42
**4**	Lorentzian	1668.9	14.69	1.98	0.09	0.17
**5**	Gaussian	1683.1	27.14	4.79	0.21	0.41
$t_{24\ h}$						
**Peak**	Peak type	Location (cm^{-1})	FWHM (cm^{-1})	Raw Area	Percent of Total Area	Percent of secondary structure content
**1**	Lorentzian	1604.1	8.28	2.42	0.09	
**2**	Gaussian	1640.5	86.59	9.69	0.38	
**3**	Gaussian	1656.9	29.78	5.28	0.21	0.39
**4**	Lorentzian	1668.2	14.3	2.7	0.11	0.20
**5**	Gaussian	1682.1	27.48	5.52	0.22	0.41

Table S4. Fits of Raman spectra shown in Fig. S4

***$t_{48\,h}$* droplets**						
**Peak**	Peak type	Location (cm^{-1})	FWHM (cm^{-1})	Raw Area	Percent Total Area	Percent secondary structure
**1**	Lorentzian	1604.7	8.31	2.26	0.09	
**2**	Gaussian	1640.5	86.59	10.34	0.41	
**3**	Gaussian	1658	30.18	4.84	0.19	0.39
**4**	Lorentzian	1668.9	15.01	3.04	0.12	0.24
**5**	Gaussian	1683.5	26.05	4.46	0.18	0.37
***$t_{48\,h}$* fibrils**						
**Peak**	Peak type	Location (cm^{-1})	FWHM (cm^{-1})	Raw Area	Percent Total Area	Percent of secondary structure
**1**	Lorentzian	1604.8	8.29	2.29	0.07	
**2**	Gaussian	1640.5	86.59	13.41	0.40	
**3**	Gaussian	1651.1	23.33	2.31	0.07	0.13
**4**	Lorentzian	1666.7	20	10.51	0.32	0.60
**5**	Gaussian	1680	23.33	4.6	0.14	0.27

Table S5. Fits of Raman spectra shown in Fig. S5

***$t_{48\ h}$* droplets**						
**Peak**	Peak type	Location (cm^{-1})	FWHM (cm^{-1})	Raw Area	Percent Total Area	Percent secondary structure
**1**	Lorentzian	1604.4	8.06	2.26	0.09	
**2**	Gaussian	1640.5	86.59	10.07	0.39	
**3**	Gaussian	1656.9	28.6	4.74	0.18	0.35
**4**	Lorentzian	1668.5	15.05	3.30	0.13	0.25
**5**	Gaussian	1682.2	27.55	5.29	0.21	0.40
***$t_{48\ h}$* fibrils**						
**Peak**	Peak type	Location (cm^{-1})	FWHM (cm^{-1})	Raw Area	Percent Total Area	Percent of secondary structure
**1**	Lorentzian	1604.5	8.19	2.23	0.07	
**2**	Gaussian	1640.5	86.59	10.44	0.35	
**3**	Gaussian	1650	21.21	1.86	0.06	0.10
**4**	Lorentzian	1666.4	21.15	10.91	0.37	0.64
**5**	Gaussian	1680	24.04	4.37	0.15	0.26

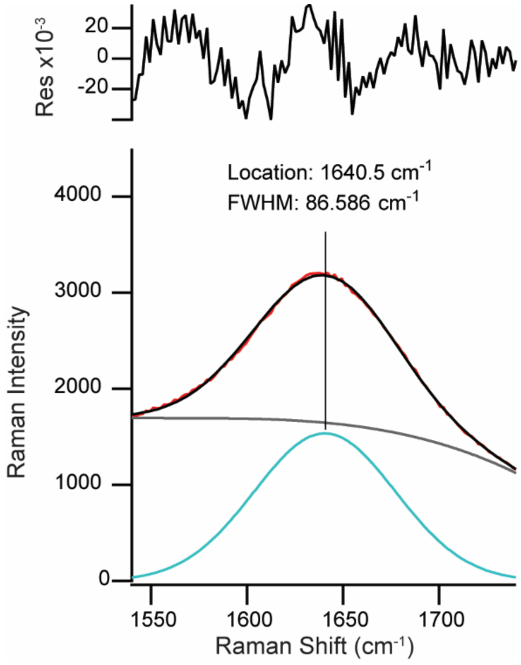

Figure S1. The bending mode of water (red curve) was adequately fit with a single Gaussian (cyan) with peak center and FWHM of 1640.5 and 86.6 cm^{-1}, respectively. Residuals are shown in the top panel. These parameters were used for all Raman spectral fitting. The solution was measured under comparable conditions at RT, containing 10 mM NaPi and 200 mM NaCl, pH 7.4.

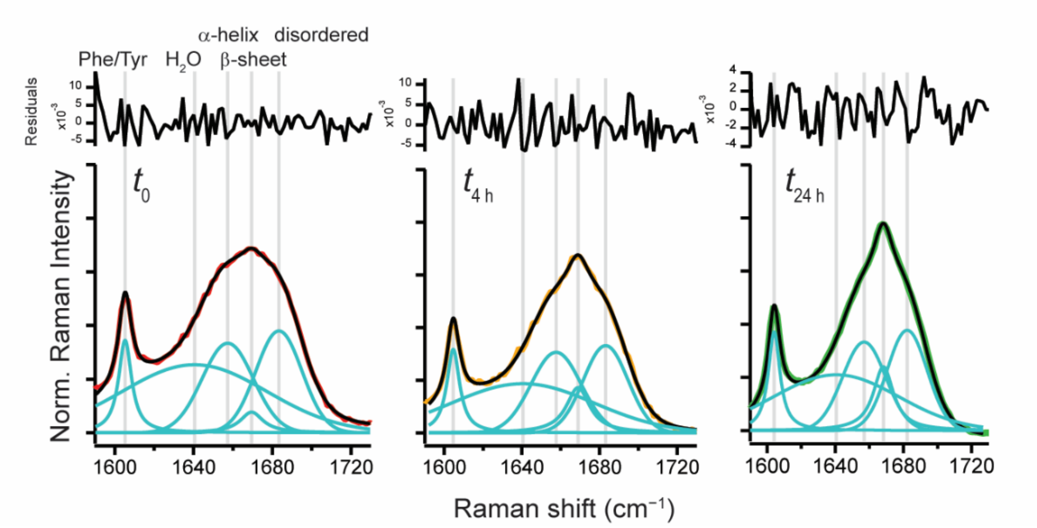

Figure S2. Additional data set and their fits. Data were averaged from at least 3 different droplets at the indicated times. Fits and individual peak components are shown in black and cyan, respectively, where the gray lines denoted peak center positions. Residuals are also shown above. Parameters can be found in **Table S3**.

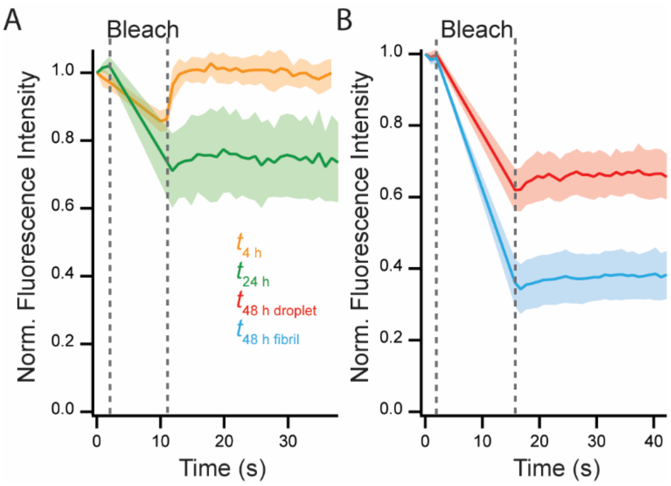

Figure S3. Average FRAP data after 4, 24, and 48 h of incubation. (A) Normalized average FRAP data from droplets at 4 (orange) and 24 h (green) h post-incubation at 22 °C. Thirty iterations at 100% laser power were used to bleach. (B) Normalized average FRAP data from droplet like structures (red) and fibrils (cyan) at 48 h post-incubation at 22 °C. Fifty iterations at 100% laser power were used to bleach. Lines and shading represent the mean and standard deviation, respectively (*n* = 5-7) ([Wfree] =100 µM in 10 mM NaPi, 200 mM NaCl, pH 7.4)

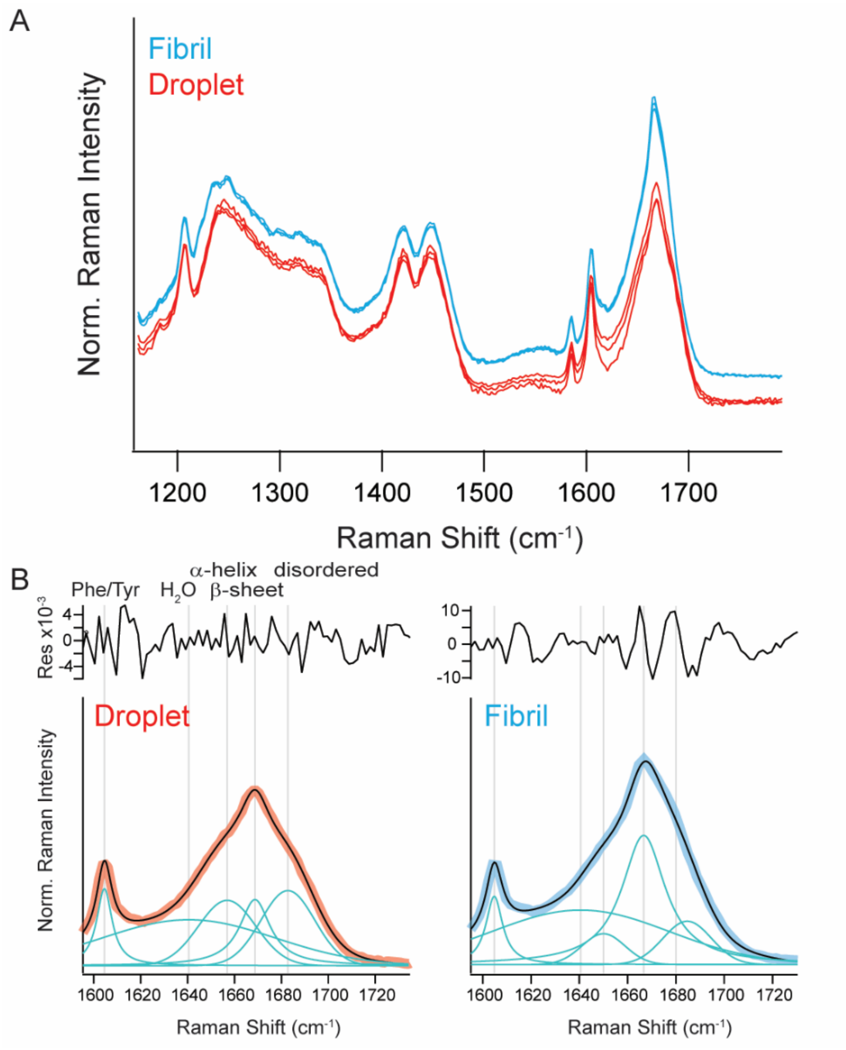

Figure S4. Full Raman spectra and amide-I fits for droplets and fibrils after 48 h of incubation. (A) Full Raman spectra of fibril (cyan) and droplet (red) locations shown in **Figure 4A**. Spectra are offset for clarity and normalized to the Phe breathing peak (1003 cm^{-1}) for comparison. (B) Fits of the amide-I band region. Data were averaged and colored as in panel A. Fits and individual peak components are shown in black and cyan, respectively, where the dashed lines denoted peak center positions. Residuals are also shown. Fit parameters can be found in **Table S4**.

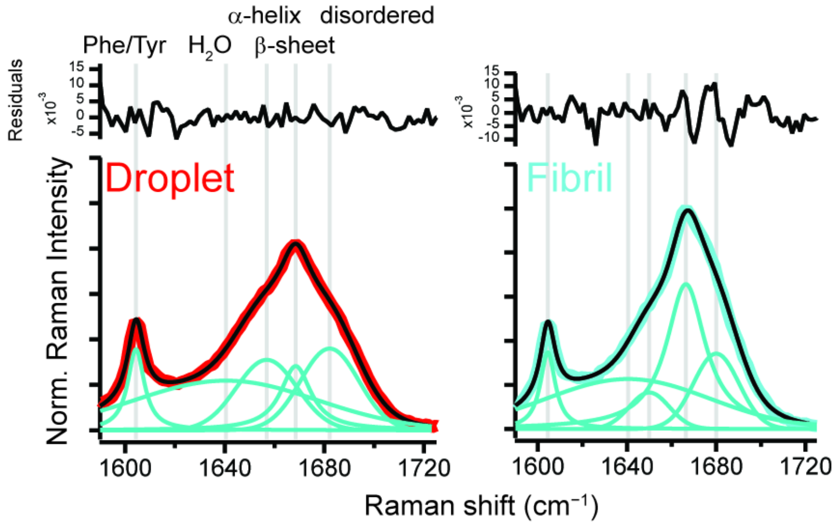

Figure S5. Additional data set and their fits. Data were averaged from at least 5 different locations for each aggregate type. Fits and individual peak components are shown in black and cyan, respectively, where the gray lines denoted peak center positions. Residuals are also shown above. Fit parameters can be found in **Table S5**
